# *PRKAR1A* deficiency impedes hypertrophy and reduces heart size

**DOI:** 10.14814/phy2.14405

**Published:** 2020-03-25

**Authors:** Yuening Liu, Peng Xia, Jingrui Chen, W. Patricia Bandettini, Lawrence S. Kirschner, Constantine A. Stratakis, Zhaokang Cheng

**Affiliations:** ^1^ Department of Pharmaceutical Sciences Washington State University Spokane WA USA; ^2^ National Heart, Lung, and Blood Institute National Institutes of Health Bethesda MD USA; ^3^ Department of Cancer Biology and Genetics The Ohio State University Columbus OH USA; ^4^ Section on Endocrinology and Genetics Eunice Kennedy Shriver National Institute of Child Health and Human Development National Institutes of Health NIH‐Clinical Research Center Bethesda MD USA

**Keywords:** adenylyl cyclase, cAMP, cardiomyopathy, catecholamine, myocardial development

## Abstract

Protein kinase A (PKA) activity is pivotal for proper functioning of the human heart, and its dysregulation has been implicated in a variety of cardiac pathologies. PKA regulatory subunit 1α (R1α, encoded by the *PRKAR1A* gene) is highly expressed in the heart, and controls PKA kinase activity by sequestering PKA catalytic subunits. Patients with *PRKAR1A* mutations are often diagnosed with Carney complex (CNC) in early adulthood, and may die later in life from cardiac complications such as heart failure. However, it remains unknown whether *PRKAR1A* deficiency interferes with normal heart development. Here, we showed that left ventricular mass was reduced in young CNC patients with *PRKAR1A* mutations or deletions. Cardiac‐specific heterozygous ablation of *PRKAR1A* in mice increased cardiac PKA activity, and reduced heart weight and cardiomyocyte size without altering contractile function at 3 months of age. Silencing of *PRKAR1A*, or stimulation with the PKA activator forskolin completely abolished α1‐adrenergic receptor‐mediated cardiomyocyte hypertrophy. Mechanistically, depletion of *PRKAR1A* provoked PKA‐dependent inactivating phosphorylation of Drp1 at S637, leading to impaired mitochondrial fission. Pharmacologic inhibition of Drp1 with Mdivi 1 diminished hypertrophic growth of cardiomyocytes. In conclusion, *PRKAR1A* deficiency suppresses cardiomyocyte hypertrophy and impedes heart growth, likely through inhibiting Drp1‐mediated mitochondrial fission. These findings provide a potential novel mechanism for the cardiac manifestations associated with CNC.

## Graphical abstract

PRKAR1A mutations in humans are known to cause Carney complex, an autosomal dominant genetic disease often associated with premature death. Here, we report that PRKAR1A deficiency suppresses cardiomyocyte hypertrophy and reduces heart size, likely through PKA‐dependent inhibition of Drp1‐mediated mitochondrial fission.

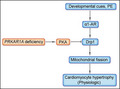

## 1 INTRODUCTION

Myocardial 3’,5’‐cyclic adenosine monophosphate (cAMP)‐dependent protein kinase (protein kinase A, PKA) is a key regulator of heart rate and cardiac contractility, through phosphorylation of multiple calcium‐handling proteins involved in excitation–contraction coupling (Zhang et al., 2019). The PKA holoenzyme consists of two regulatory and two catalytic subunits. When intracellular cAMP levels are low, the regulatory subunits bind and inhibit the catalytic subunits. Stimulation of β‐adrenergic receptors (β‐ARs) by the catecholamine neurohormones results in activation of the stimulatory G protein (Gαs), thereby initiating adenylyl cyclase‐dependent synthesis of cAMP. The cAMP molecules then bind the PKA regulatory subunits, leading to release of the PKA catalytic subunits and subsequent kinase activation.

Mammalian cells express four isoforms of PKA regulatory subunits that are functionally nonredundant: R1α, R1β, R2α, and R2β (encoded by *PRKAR1A*, *PRKAR1B, PRKAR2A*, and *PRKAR2B*, respectively). Among these regulatory subunits, R1α is the most abundantly and ubiquitously expressed, and is the only isoform required for embryonic heart development (Kirschner, Yin, Jones, & Mahoney, 2009; Taylor, Ilouz, Zhang, & Kornev, 2012). In humans, inactivating mutations or deletions of the *PRKAR1A* gene have been associated with Carney complex (CNC), an autosomal dominant genetic disorder characterized by spotty skin pigmentation and tumors in the endocrine glands, heart (cardiac myxoma), skin, breast, and other body parts (Casey et al., 2000; Kirschner et al., 2000). CNC is typically diagnosed from childhood to adulthood, with a median age of detection at approximately 20 years (Correa, Salpea, & Stratakis, 2015). The historic adjusted average life span for these patients is 50–55 years (Correa et al., 2015). It is estimated that more than 50% of mortality related to CNC is attributed to cardiovascular complications such as heart failure (Correa et al., 2015), indicating a potential role of *PRKAR1A* in the maintenance of heart function and morphology. In this study, we evaluated the heart size in a cohort of young CNC patients with identified *PRKAR1A* mutations or deletions. Our analysis revealed that young patients with CNC had reduced left ventricular mass.

CNC is predominantly associated with *PRKAR1A* haploinsufficiency in patients (Casey et al., 2000; Veugelers et al., 2004). To investigate the impact of *PRKAR1A* deficiency on myocardial development, we generated a cardiac‐specific heterozygous *PRKAR1A* knockout mouse line (c*PRKAR1A* ^+/‐^). Here, we showed that ablation of *PRKAR1A* in mice diminished cardiomyocyte hypertrophy and impeded physiological heart growth. In vitro studies further revealed that disruption of *PRKAR1A* induced PKA‐dependent Drp1 inhibition, which was sufficient to repress hypertrophy.

## 2 METHODS

### 2.1 Human studies

#### 2.1.1 Patients

CNC patients with *PRKRA1A* mutations or deletions were recruited to an ongoing study at the National Institutes of Health Clinical Center, under a protocol approved by the Institutional Review Board (Correa et al., 2015; Kirschner et al., 2000).

#### 2.1.2 Magnetic resonance imaging (MRI) in CNC patients

Cardiovascular magnetic resonance steady‐state free precession cine imaging was performed as described previously (Kramer et al. 2013), in a small group of CNC patients with identified *PRKRA1A* mutations or deletions but without prior history of cardiac myxoma. To avoid the confounders of hypertrophy that may occur with age and hypertension, the subjects selected were below or at 21 years of age. Endocardial and epicardial borders were manually drawn on volumetric short‐axis slices of the left ventricle to determine the end‐diastolic myocardial mass. The left ventricular mass data were then indexed to body surface area, and compared with age‐ and gender‐matched normal references (Ven et al., 2020).

### 2.2 Animal studies

#### 2.2.1 Animals

*PRKAR1A* ^flox/+^ mice (Kirschner et al., 2005) and *Mlc2v‐Cre* ^+/‐^ mice (Chen et al., 1998) were backcrossed to the C57BL/6 background for at least eight generations prior to subsequent breeding. All animals were housed in the campus vivarium accredited by the American Association for Accreditation of Laboratory Animal Care. Animal studies comply with the National Institutes of Health Guide for the Care and Use of Laboratory Animals (8th edition, 2011), and were approved by the Institutional Animal Care and Use Committee at Washington State University.

#### 2.2.2 Genotyping

Genotyping PCR was performed using DNA isolated from tail snips, hearts, and other tissues as described previously with minor modification (Chen et al., 1998; Kirschner et al., 2005). The PCR amplification protocol was 35 cycles of 30 s at 95°C, 30 s at 58°C, and 30 s at 72°C. The primer sequences for Cre were as follows: 5′ GTT CGC AAG AAC CTG ATG GAC A‐3′ and 5′‐CTA GAG CCT GTT TTG CAC GTT C‐3′ (*Cre* ^+^ product: 340 bp). To distinguish wild‐type and floxed *PRKAR1A* alleles, the following primers were used: 5′‐GCA GGC GAG CTA TTA GTT TA‐3′ and 5′‐CAT CCA TCT CCT ATC CCC TTT‐3′ (*PRKAR1A* ^flox^ product: 350 bp; *PRKAR1A* ^+^ product: 240 bp). The primers used to detect *PRKAR1A* deletion were as follows: 5′‐CAA GCT AGC TTG GCT GGA CGT A‐3′ and 5′‐CAT CCA TCT CCT ATC CCC TTT‐3′ (*PRKAR1A* ^‐^ product: 175 bp).

#### 2.2.3 Echocardiography

Cardiac contractile function and morphology in 3‐month‐old male mice was evaluated by echocardiography using the VisualSonics ultrasound imaging system VEVO 2100 equipped with a 55‐MHz MS550S transducer. All mice were placed on a warmed table under anesthesia with 1.5% isoflurane for imaging. Standard short‐axis M‐mode views were recorded at heart rate between 450 and 600 beats per minute. Functional parameters were averaged from three consecutive contractions.

#### 2.2.4 Measurement of heart weight and cardiomyocyte cross‐sectional area

Hearts from 3‐month‐old male mice were harvested, weighed, and immediately fixed in 4% paraformaldehyde. Heart paraffin sections were subjected to antigen retrieval using 10 mmol/L citrate buffer (pH 6.0) prior to staining with wheat germ agglutinin (W32464, Invitrogen) and mouse anticardiac troponin T (MS‐295‐P, Thermo Scientific, 1:100). Cardiomyocyte cross‐sectional area was measured from at least 250 cells in 8–9 fields per heart using the ImageJ software.

### 2.3 In vitro studies

#### 2.3.1 Cell culture

Neonatal rat cardiomyocytes (NRCMs) were prepared from 2‐ to 4‐day‐old Sprague–Dawley rats (Envigo) according to published protocols (Xia et al., 2018). Briefly, rat hearts were cut into small pieces (~1 mm^3^), digested with trypsin (50 μg/ml), and then collagenase (100 units/ml). Cells were resuspended in medium 199 supplemented with 15% fetal bovine serum, plated on surfaces precoated with 0.2% gelatin, and then cultured in serum‐free medium 199. H9c2 myoblasts derived from rat heart tissue (CRL‐1446, ATCC) were maintained in Dulbecco's modified Eagle's medium supplemented with 10% fetal bovine serum.

#### 2.3.2 siRNA transfection

NRCMs were transfected with siRNAs in serum‐free medium 199 using HiPerfect transfection reagent (Qiagen), and H9c2 cells were transfected in serum‐free Dulbecco's modified Eagle's medium using DharmaFECT 1 transfection reagent (Dharmacon) according to the manufacturers’ protocols. The siRNA sequences used were as follows: *PRKAR1A* siRNAs (siPRKAR1A), GACAGAUUCAGAGCCUACA[dT][dT]; and control GFP siRNAs (siGFP), GGUGCGCUCCUGGACGUAGCC[dT][dT].

#### 2.3.3 Western blotting

Animal tissues or cells were lysed in radioimmune precipitation assay buffer supplemented with protease and phosphatase inhibitors (Thermo Scientific). Protein lysates were subjected to sodium dodecyl sulfate polyacrylamide gel electrophoresis, followed by transfer onto polyvinylidene difluoride membranes. The following antibodies were used for immunoblotting: rabbit anti‐PKA R1α (ab139695, Abcam, 1:1000), rabbit anti‐phospho‐PKA substrate (RRXS*/T*, #9624, Cell Signaling, 1:1000), rabbit anti‐phospho‐Drp1 (S637, #4867, Cell Signaling,1:1000), rabbit anti‐GAPDH (sc‐25778, Santa Cruz Biotechnology, 1:1000), and mouse anti‐β‐actin (sc‐47778, Santa Cruz Biotechnology, 1:1000).

#### 2.3.4 Immunofluorescence

Immunostaining was performed as described previously (Cheng et al., 2017), with the following primary antibodies: mouse anti‐cardiac troponin T (MS‐295‐P, Thermo Scientific, 1:100), mouse anti‐COX IV (#11967, Cell Signaling, 1:100), or rabbit anti‐Drp1 (#8570, Cell Signaling, 1:100). Cardiomyocyte cell surface area was measured using ImageJ. Manders’ coefficient for evaluation of colocalization was analyzed using the ImageJ plugin Coloc 2.

#### 2.3.5 Mitochondrial morphology analysis

Cells were incubated with MitoTracker Red for 30 min and visualized under a confocal laser‐scanning microscope (Leica). Images acquired from three independent experiments were analyzed using the ImageJ software. To calculate the percentage of cells with elongated or fragmented mitochondria, at least 500 cells were counted. To analyze mitochondrial length, at least 15 mitochondrial particles per cell from 40 cells were measured.

#### 2.3.6 Statistical analysis

Statistical analysis was performed using GraphPad Prism 7.02. Results are expressed as mean ± *SEM* unless indicated otherwise. Student's unpaired *t* test was used to compare values between two groups except the MRI data from CNC patients, which were analyzed using Mann‐Whitney test. One‐way analysis of variance followed by Tukey post hoc test was used for multiple‐group comparisons. A value of *p* < .05 was considered statistically significant.

## 3 RESULTS

### 3.1 Young CNC patients with *PRKAR1A* mutations or deletions had reduced left ventricular mass

Mutations or deletions of the *PRKAR1A* gene cause CNC in humans (Correa et al., 2015). The major clinical manifestation of CNC in the heart is cardiac myxoma (Correa et al., 2015). To determine if CNC is associated with abnormal heart growth, we measured left ventricular mass in CNC patients with identified *PRKAR1A* mutations or deletions. As left ventricular mass is profoundly increased by hypertension, which is more prevalent at older ages (Buford, 2016), all included patients were at or below 21 years of age (mean age: 15.91 ± 3.30 years). When indexed to body surface area, left ventricular masses in CNC patients were significantly smaller than those in age‐ and gender‐matched reference controls (Table 1).

**Table 1:** Left ventricular mass indexed to body surface area in young patients diagnosed with Carney complex (CNC), with identified *PRKAR1A* mutations or deletions

	Reference control (g/m^2^)	CNC (g/m^2^)	*N*	*p* value
All	59.29 ± 8.73	49.02 ± 9.35	17	.0031 *
Male	65.28 ± 9.69	51.78 ± 11.37	8	.011 *
Female	53.96 ± 1.49	46.56 ± 6.86	9	.0046 *

Note

Data are mean ± standard deviation. Mann–Whitney test.

* *p* < .05 between reference control and CNC. The young CNC patients had a mean age of 15.91 ± 3.30 years, and were compared with age‐ and gender‐matched reference controls (Ven et al., 2020).

### 3.2 Generation of cardiac‐specific *PRKAR1A* heterozygous knockout mice

**Figure 1:**
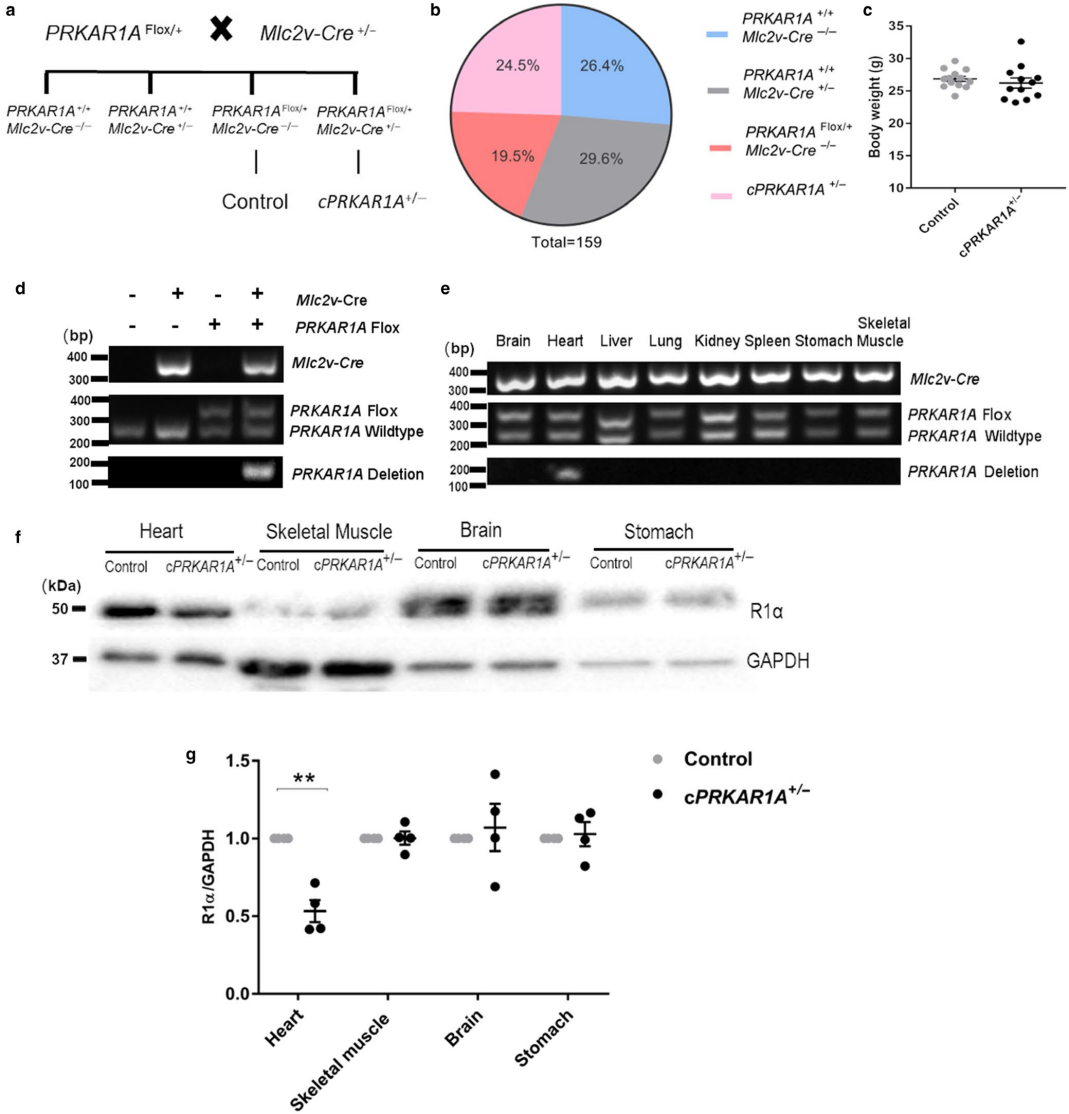
Generation of cardiac‐specific *PRKAR1A* heterozygous knockout mice. (a) Breeding strategy for cardiac‐specific *PRKAR1A* heterozygous knockout (*cPRKAR1A* ^+/‐^) mice: *PRKAR1A* ^Flox/+^/ *Mlc2v‐Cre* ^+/‐^. (b) Neonatal *cPRKAR1A* ^+/‐^ mice and littermates were born with the expected Mendelian ratio from a total of 159 mice. (c) Body weight of 3‐month‐old male *cPRKAR1A* ^+/‐^ and *PRKAR1A* ^Flox/+^ control mice (Control, *n* = 13; *cPRKAR1A* ^+/‐^, *n* = 12). (d) DNA isolated from control or *cPRKAR1A* ^+/‐^ heart (ventricle) was used for genotyping PCR. *PRKAR1A* deletion band was only detected in the *cPRKAR1A* ^+/‐^ heart. (e) DNA isolated from heart (ventricle), skeletal muscle, brain, and stomach of the *cPRKAR1A* ^+/‐^ mouse was used for genotyping PCR. *PRKAR1A* deletion band was only detected in the heart, but not in other tissues of the *cPRKAR1A* ^+/‐^ mouse. (f,g) Heart protein lysates from 3‐month‐old male mice were subjected to immunoblotting (*n* = 4 per group). Values are mean ± *SEM* and analyzed using two‐tailed Student's *t* test. ** *p* < .01

To determine if *PRKAR1A* deficiency delays heart growth, we generated cardiac‐specific *PRKAR1A* heterozygous knockout (*PRKAR1A* ^flox/+^/*Mlc2v‐Cre* ^+/‐^, hereafter, referred to as *cPRKAR1A* ^+/‐^) mice using the Cre‐loxP technology, by crossing the *PRKAR1A* ^flox/+^ mice with the *Mlc2v‐Cre* ^+/‐^ mice (Figure 1a). Neonatal *cPRKAR1A* ^+/‐^ mice were born with the expected Mendelian ratio (Figure 1b), and developed into adulthood with similar survival rate as littermates. As the *PRKAR1A* ^flox/+^ and *Mlc2v‐Cre* ^+/‐^ littermates displayed comparable left ventricular mass and contractile function (Figure S1), only the *PRKAR1A* ^flox/+^ line was used as control thereafter (Figure 1a). Adult *cPRKAR1A* ^+/‐^ mice were indistinguishable from control mice in physical appearance, and had similar body weight as controls (Figure 1c). *PRKAR1A* gene recombination was only detected in *cPRKAR1A* ^+/‐^ mouse heart, but not in control hearts from the other three genotypes (Figure 1d), or noncardiac tissues in the *cPRKAR1A* ^+/‐^ mouse (Figure 1e), indicating cardiac‐specific *PRKAR1A* ablation. Immunoblotting analysis of tissue lysates extracted from 3‐month‐old *cPRKAR1A* ^+/‐^ mice further revealed a ~ 50% decrease in PKA R1α protein level in the heart, but not in the skeletal muscle, brain, or stomach (Figure 1f,g).

### 3.3 Disruption of *PRKAR1A* induced PKA activation without altering cardiac function

**Figure 2:**
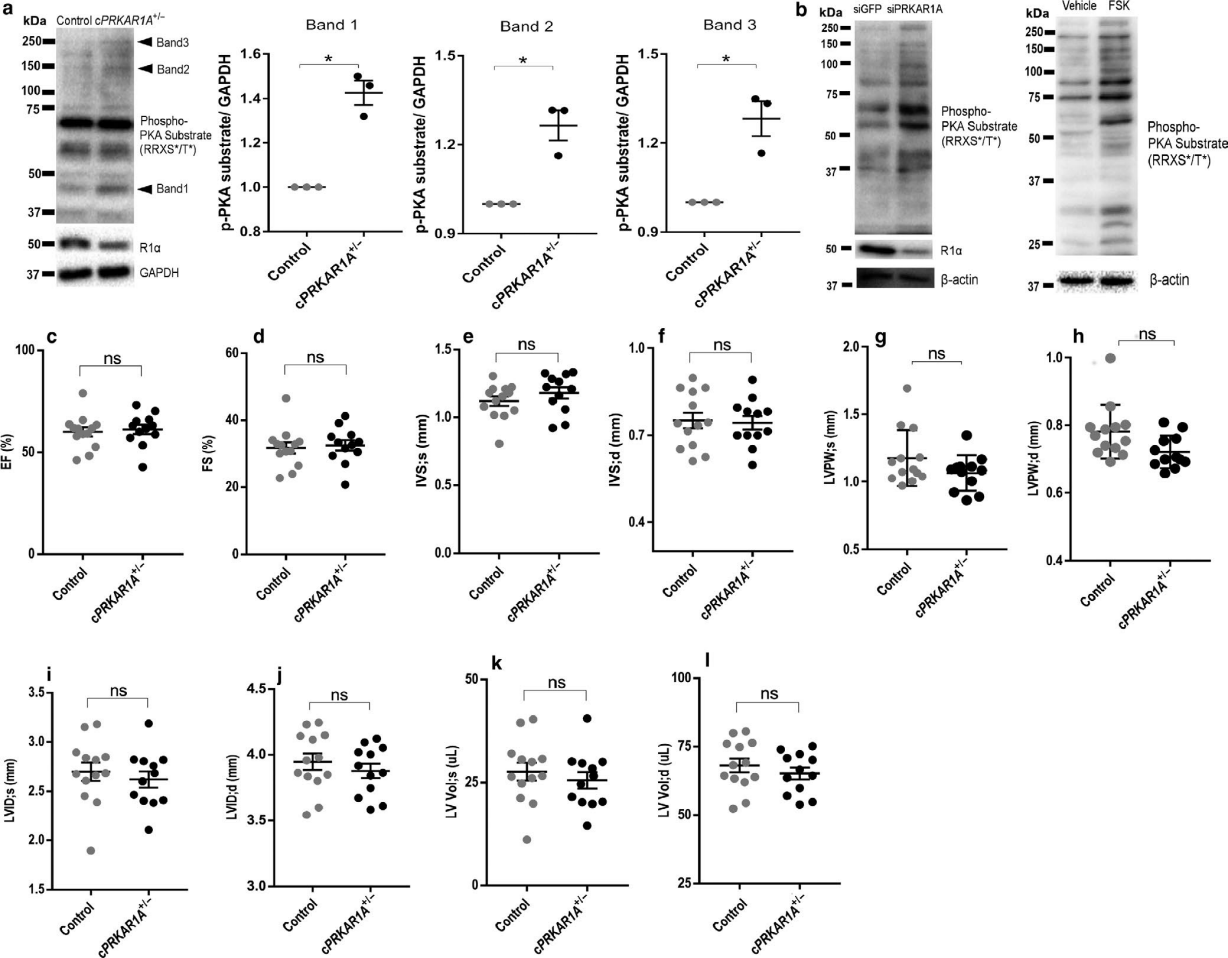
Disruption of *PRKAR1A* induced PKA activation without altering cardiac function. (a) Western blot analysis of control and *cPRKAR1A* ^+/‐^ heart protein lysates extracted from 3‐month‐old male mice. PKA activity was assessed using phospho‐PKA Substrate (RRXS*/T*) antibody, and GAPDH served as a loading control. Arrowheads indicate elevated levels of phosphorylated PKA substrates (bands 1–3) in *cPRKAR1A* ^+/‐^ heart. (b) NRCMs were transfected with control (siGFP) or *PRKAR1A* siRNA (siPRKAR1A) for 48h. PKA activity was assessed using phospho‐PKA Substrate (RRXS*/T*) antibody, with β‐actin as a loading control. NRCMs incubated with the adenylyl cyclase agonist forskolin (FSK, 10 µM) for 24 h served as a positive control for PKA activation. (c‐l) Cardiac function was assessed using echocardiography (Control, *n* = 13; *cPRKAR1A* ^+/‐^, *n* = 12). (c,d) Left ventricular EF (ejection fraction) and FS (fractional shortening). (e,f) IVS;s (interventricular septal thickness at end systole), IVS;d (interventricular septal thickness at end diastole). (g,h) LVPW;s (left ventricular posterior wall thickness at end systole), LVPW;d (left ventricular posterior wall thickness at end diastole). (i,j) LVID;s (left ventricular internal dimension at end systole), LVID;d (left ventricular internal dimension at end diastole). (k,l) LV,vol;s (left ventricular volume at end systole), LV,vol;d (left ventricular volume at end diastole). Values are mean ± *SEM* and analyzed using two‐tailed Student's *t* test. * *p* < .05; ns, not significant

In agreement with previous findings that homozygous ablation of *PRKAR1A* causes PKA activation (Amieux et al., 2002; Yin et al., 2008), measurement of PKA activity using a phospho‐PKA substrate (RRXS*/T*) antibody revealed enhanced phosphorylation of some, but not all PKA substrates in the *cPRKAR1A* ^+/‐^ heart (Figure 2a). Moreover, transfection of neonatal rat cardiomyocytes (NRCMs) with *PRKAR1A*‐specific siRNAs resulted in a similar decrease (~50%) in the level of R1α protein, and markedly increased the levels of phosphorylated PKA substrates (Figure 2b). Robust increases in phospho‐PKA substrate levels were also detected after incubation with forskolin, an adenylyl cyclase agonist known to induce extensive PKA activation (Figure 2b). At 3 months of age, *cPRKAR1A* ^+/‐^ and control mice displayed comparable left ventricular ejection fraction (EF, Figure 2c), fractional shortening (FS, Figure 2d), and interventricular septum thicknesses at both end systole and end diastole (IVS, Figure 2e,f). Interestingly, heterozygous deletion of *PRKAR1A* resulted in slight but nonsignificant reductions in left ventricular posterior wall thicknesses (LVPW, Figure 2g,h), left ventricular internal dimension (LVID, Figure 2i,j), and left ventricular volume (LV Vol, Figure 2k,l). Together, these results suggested that basal contractile function was maintained in *cPRKAR1A* ^+/‐^ mice.

### 3.4 *PRKAR1A* deficiency impeded hypertrophic growth during heart development

**Figure 3:**
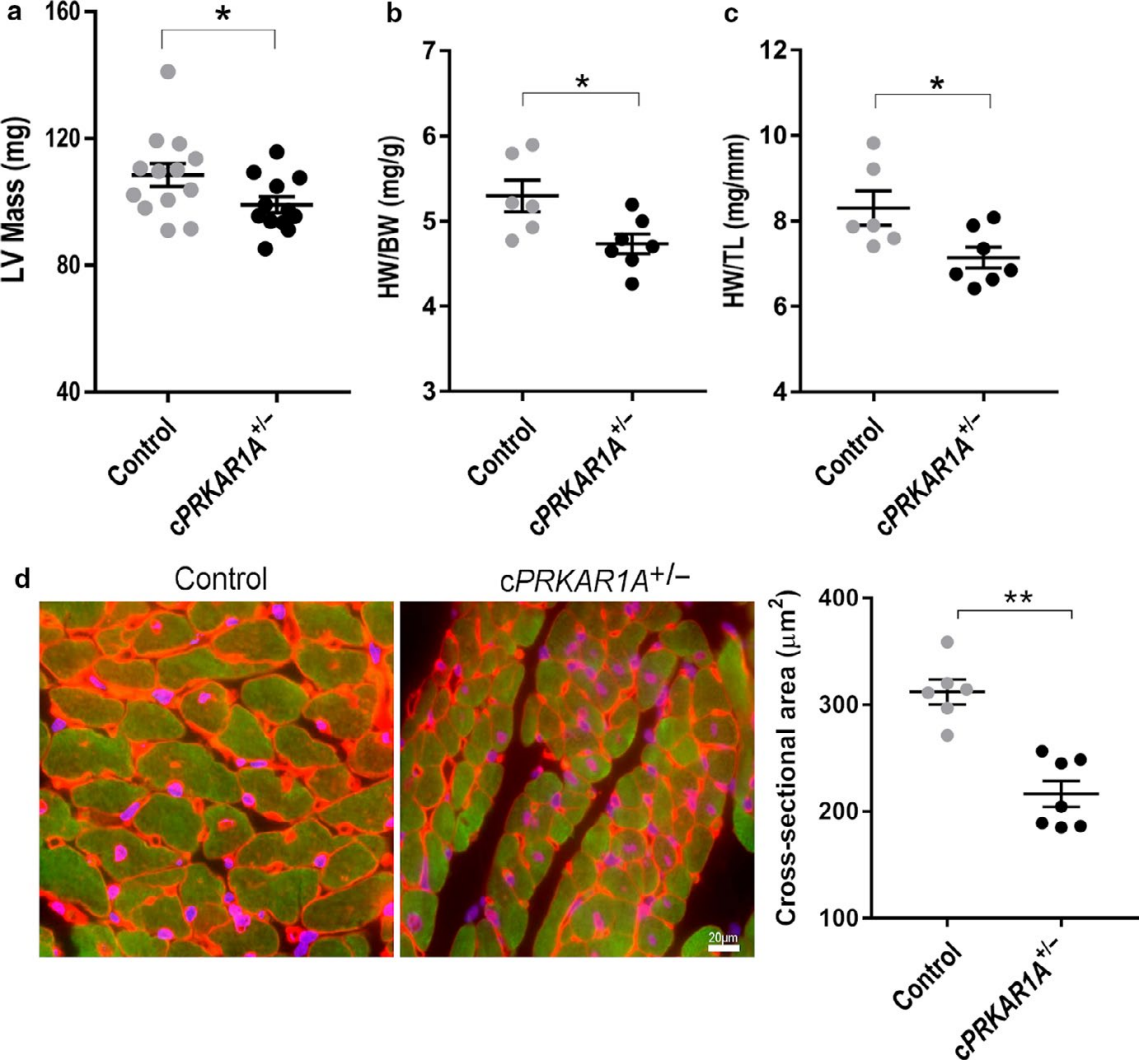
*PRKAR1A* deficiency impeded hypertrophic growth during heart development. (a) Left ventricular (LV) mass was assessed using echocardiography (Control, *n* = 13; *cPRKAR1A* ^+/‐^, *n* = 12). (b) Heart weight to body weight ratio (Control, *n* = 6; *cPRKAR1A* ^+/‐^, *n* = 7). (c) Heart weight to tibia length ratio (Control, *n* = 6; *cPRKAR1A* ^+/‐^, *n* = 7). (d) Cardiomyocyte cross‐sectional area was analyzed by staining with wheat germ agglutinin (WGA, *red*), cardiomyocyte marker cardiac troponin T (cTnT, *green*), and nuclei (DAPI, *blue*). At least 250 cardiomyocytes were measured per group (Control, *n* = 6; *cPRKAR1A* ^+/‐^, *n* = 7). Scale bar = 20 µm. Values are mean ± *SEM* and analyzed using two‐tailed Student's *t* test. * *p* < .05, ** *p* < .01

In line with a trend toward decreased posterior wall thickness and ventricular diameter, echocardiography revealed a significant reduction in the left ventricular mass in *cPRKAR1A* ^+/‐^ mice (Figure 3a). Moreover, *PRKAR1A* ablation significantly decreased heart weight/body weight ratio and heart weight/tibia length ratio at 3 months of age (Figure 3b,c). Increases in heart weight after birth are achieved predominantly through an increase in cell size (i.e., cardiomyocyte hypertrophy) (Ahuja, Sdek, & MacLellan, 2007). To determine if *PRKAR1A* deletion hinders cardiac hypertrophic growth, heart sections of *cPRKAR1A* ^+/‐^ mice and littermates were subjected to wheat germ agglutinin (WGA) staining. As shown in Figure 3d, heterozygous deletion of *PRKAR1A* dramatically reduced cardiomyocyte cross‐sectional area, indicating diminished cardiomyocyte hypertrophy.

### 3.5 PKA activation inhibited α1‐adrenergic receptor‐mediated hypertrophy

**Figure 4:**
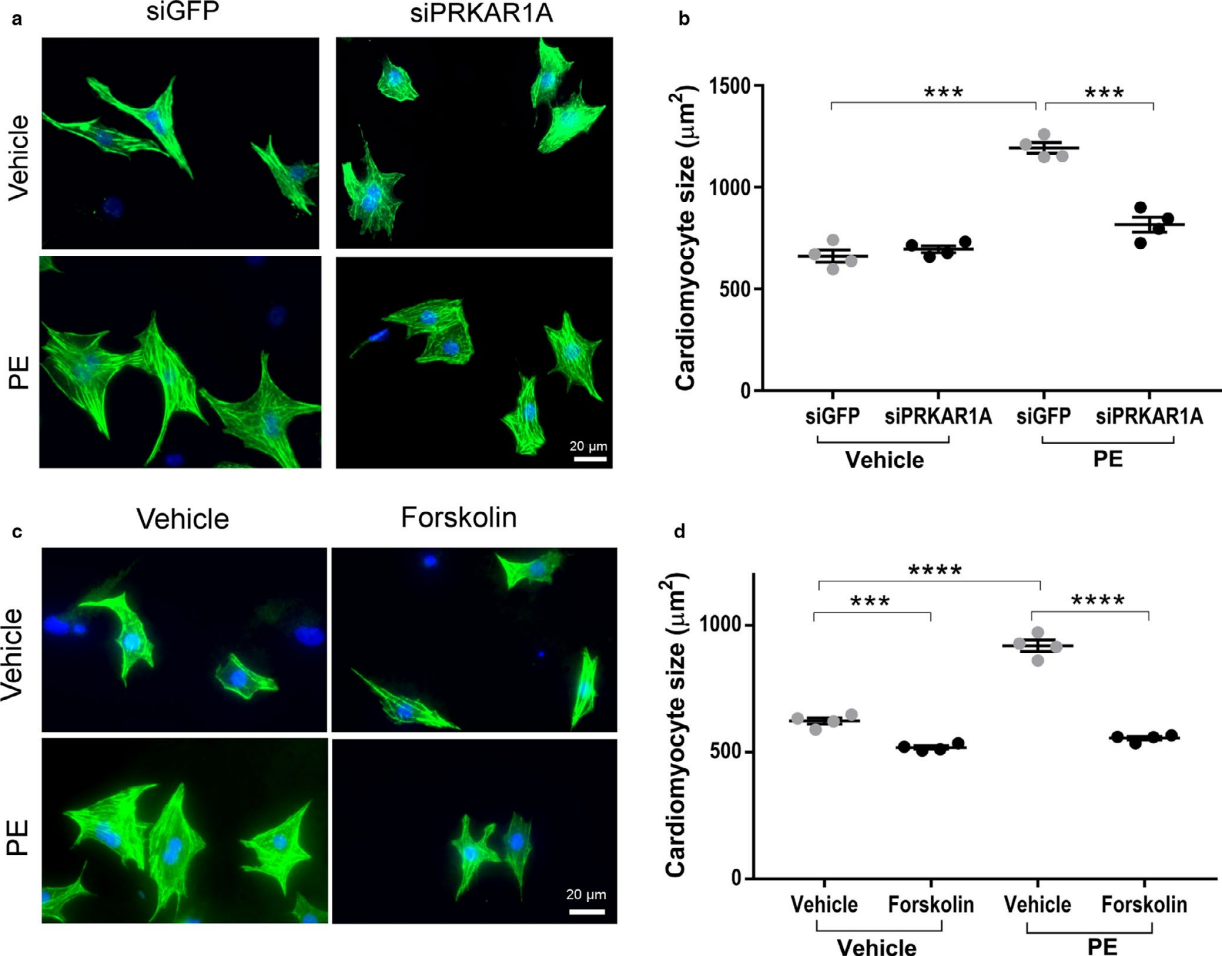
PKA activation inhibited α1‐adrenergic receptor‐mediated hypertrophy. (a,b) NRCMs were transfected with control (siGFP) or *PRKAR1A* siRNA (siPRKAR1A) prior to incubation with the α1‐adrenergic receptor agonist phenylephrine (PE, 50 µM) for 48 h. (a) Immunofluorescence staining for cardiac troponin T (cTnT, *green*) and nuclei (DAPI, *blue*) in NRCMs. Scale bar = 20µm; (b) Cardiomyocyte cell surface area was analyzed using ImageJ. (c,d) NRCMs were incubated with PE (50 µM) for 48 h in the presence of the adenylyl cyclase agonist forskolin (10 µM). (c) Immunofluorescence staining for cTnT (*green*) and nuclei (*blue*). Scale bar = 20 µm; (d) Cardiomyocyte cell surface area was analyzed using ImageJ. At least 250 cardiomyocytes were measured per group (*n* = 4). Two‐way ANOVA with Sidak test. ***p < .001; ****p < .0001

Postnatal heart growth occurs primarily via physiologic hypertrophy, which is characterized by an increase in myocyte size without causing apoptosis or fibrosis, through activation of the α1‐adrenergic receptors (O'Connell et al., 2003; O'Connell, Jensen, Baker, & Simpson, 2014). To determine if *PRKAR1A* is necessary for α1‐adrenergic receptor‐mediated hypertrophy, NRCMs were transfected with *PRKAR1A* siRNAs prior to incubation with phenylephrine (PE), a selective α1‐adrenergic receptor agonist. While PE stimulation dramatically increased myocyte surface area in control cells, *PRKAR1A*‐depleted cells were largely irresponsive to PE treatment (Figure 4a,b), suggesting that silencing of *PRKAR1A* abrogated hypertrophic growth of cardiomyocytes. To further investigate the role of PKA in hypertrophy, NRCMs were treated with PE in the presence of the PKA activator forskolin. Stimulation with forskolin completely abolished PE‐induced increase in myocyte size (Figure 4c,d). Intriguingly, treatment with forskolin alone slightly but significantly reduced cell surface area (Figure 4c,d). These results suggested that activation of PKA antagonized α1‐adrenergic receptor‐mediated cardiomyocyte hypertrophy.

### 3.6 Silencing of *PRKAR1A* resulted in mitochondrial elongation

**Figure 5:**
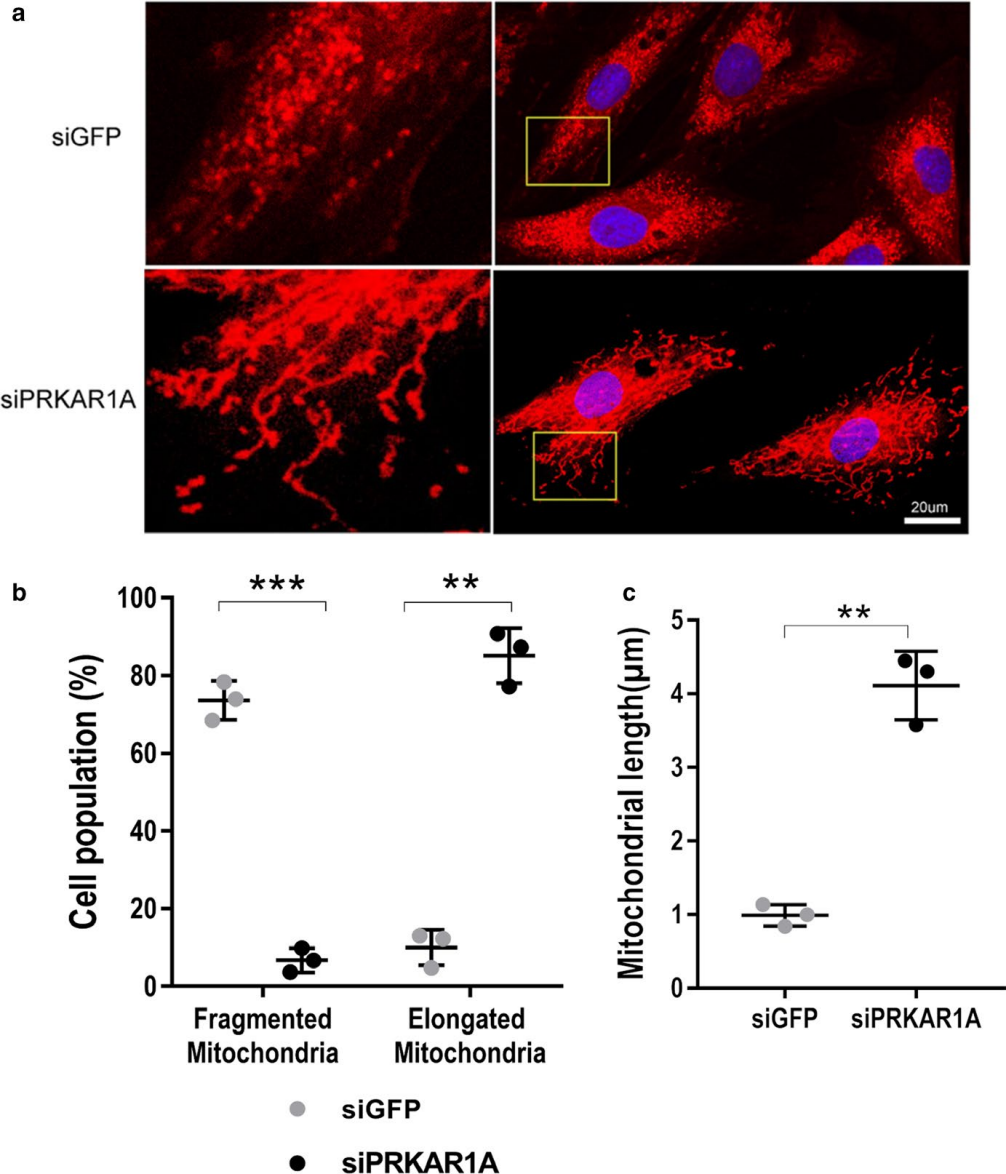
Silencing of *PRKAR1A* resulted in mitochondrial elongation. (a) H9c2 myoblasts were transfected with control (siGFP) or *PRKAR1A* siRNA (siPRKAR1A), followed by staining with MitoTracker red to label mitochondria (*Red*) and DAPI to label nuclei (*blue*). Boxed areas are enlarged on the left. Scale bar = 20µm; (b) Percentage of H9c2 cells with fragmented or elongated mitochondria. At least 500 cells were counted (*n* = 3). Two‐way ANOVA with Sidak test. (c) Mitochondrial length. At least 15 mitochondrial particles of 40 cells were measured (*n* = 3). Two‐tailed Student's *t* test. ** *p* < .01, *** *p* < .001

Initiation of cardiomyocyte hypertrophy requires mitochondrial fission, which generates fragmented mitochondria (Pennanen et al., 2014). Therefore, we examined mitochondrial morphology in *PRKAR1A*‐depleted H9c2 myoblasts. Knockdown of *PRKAR1A* dramatically reduced the number of cells with fragmented mitochondria, and robustly increased those with elongated mitochondria (Figure 5a,b). Moreover, mitochondrial length was significantly increased in *PRKAR1A*‐depleted cells (Figure 5c).

### 3.7 Knockdown of *PRKAR1A* provoked PKA‐dependent phosphorylation and inactivation of the mitochondrial fission protein Drp1

**Figure 6:**
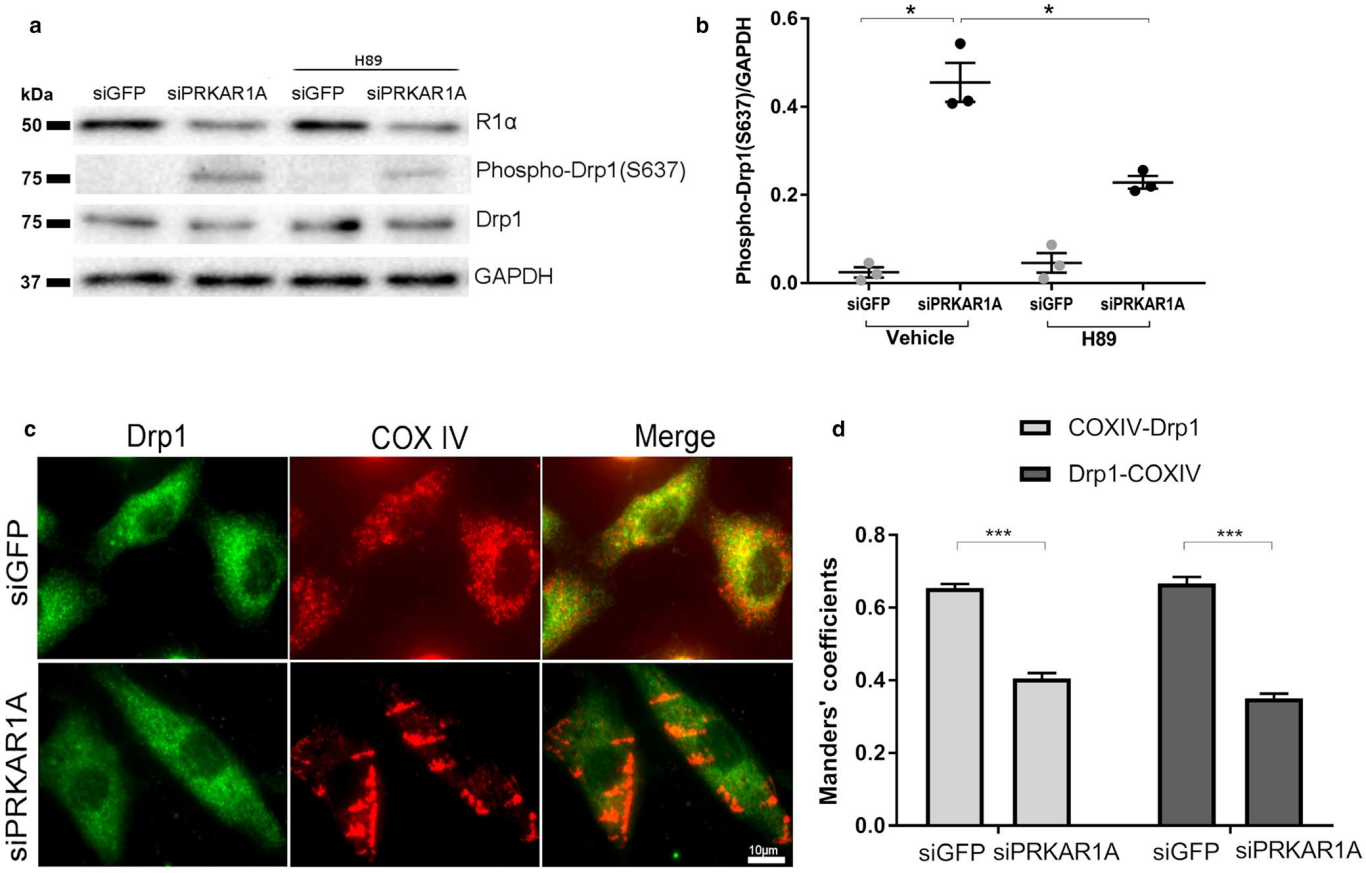
Knockdown of *PRKAR1A* provoked PKA‐dependent phosphorylation and inactivation of the mitochondrial fission protein Drp1. (a,b) NRCMs were transfected with control (siGFP) or *PRKAR1A* siRNA (siPRKAR1A) for 48h, then treated with PKA inhibitor (H89, 5µM) or vehicle for 4h. Cell lysates were immunoblotted using indicated antibodies with β‐actin as a loading control. (a) Representative images; (b) Quantitation of phospho‐Drp1 levels. Two‐way ANOVA with Sidak test. * *p* < .05; (c) NRCMs transfected with siGFP or siPRKAR1A were subjected to immunofluorescent staining for Drp1 (*green*) and the mitochondrial marker COX IV (*red*). Mitochondrial and cytosolic localized Drp1 appears yellow and green, respectively, in the merged image. Scale bar = 10µm. (d) Manders’ overlap coefficients for COX IV and Drp1. Light gray bars indicate COX IV‐associated Drp1, and dark gray bars indicate Drp1‐associated COX IV. At least 45 cells were evaluated (*n* = 4). Two‐tailed Student's *t* test. *** *p* < .001

Mitochondria are highly dynamic organelles that undergo continuous fusion and fission, and the balance between these two opposing processes determines the final mitochondrial morphology. In particular, mitochondrial fission is driven by dynamin‐related protein 1 (Drp1), a dynamin‐like GTPase that is recruited to mitochondria upon activation (Fonseca, Sanchez‐Guerrero, Milosevic, & Raimundo, 2019). Interestingly, Drp1 is phosphorylated by PKA at S637, resulting in Drp1 inactivation (Chang & Blackstone, 2007; Cribbs & Strack, 2007). In cultured cardiomyocytes, phospho‐Drp1 (S637) was not detectable at basal conditions (Figure 6a), suggesting that Drp1 exists in its active form. Intriguingly, depletion of *PRKAR1A* induced robust, spontaneous Drp1 phosphorylation at S637, which was attenuated by treatment with the small‐molecule PKA inhibitor H89 (Figure 6a,b). To further examine the effect of *PRKAR1A* depletion on Drp1 function, NRCMs were costained for Drp1 and COX IV, a mitochondrial marker protein located on the mitochondrial inner membrane. In control transfected cells, Drp1 was largely colocalized with COX IV (Figure 6c,d), indicating that Drp1 was recruited to the mitochondria to mediate fission. Remarkably, silencing of *PRKAR1A* dramatically reduced mitochondrial Drp1, resulting in predominant cytosolic localization of Drp1 (Figure 6c,d). Collectively, these results suggested that *PRKAR1A* deficiency provoked Drp1 S637 phosphorylation through activation of PKA, resulting in suppression of Drp1‐mediated mitochondrial fission.

### 3.8 Inhibition of Drp1 attenuated α1‐adrenergic receptor‐mediated hypertrophy

**Figure 7:**
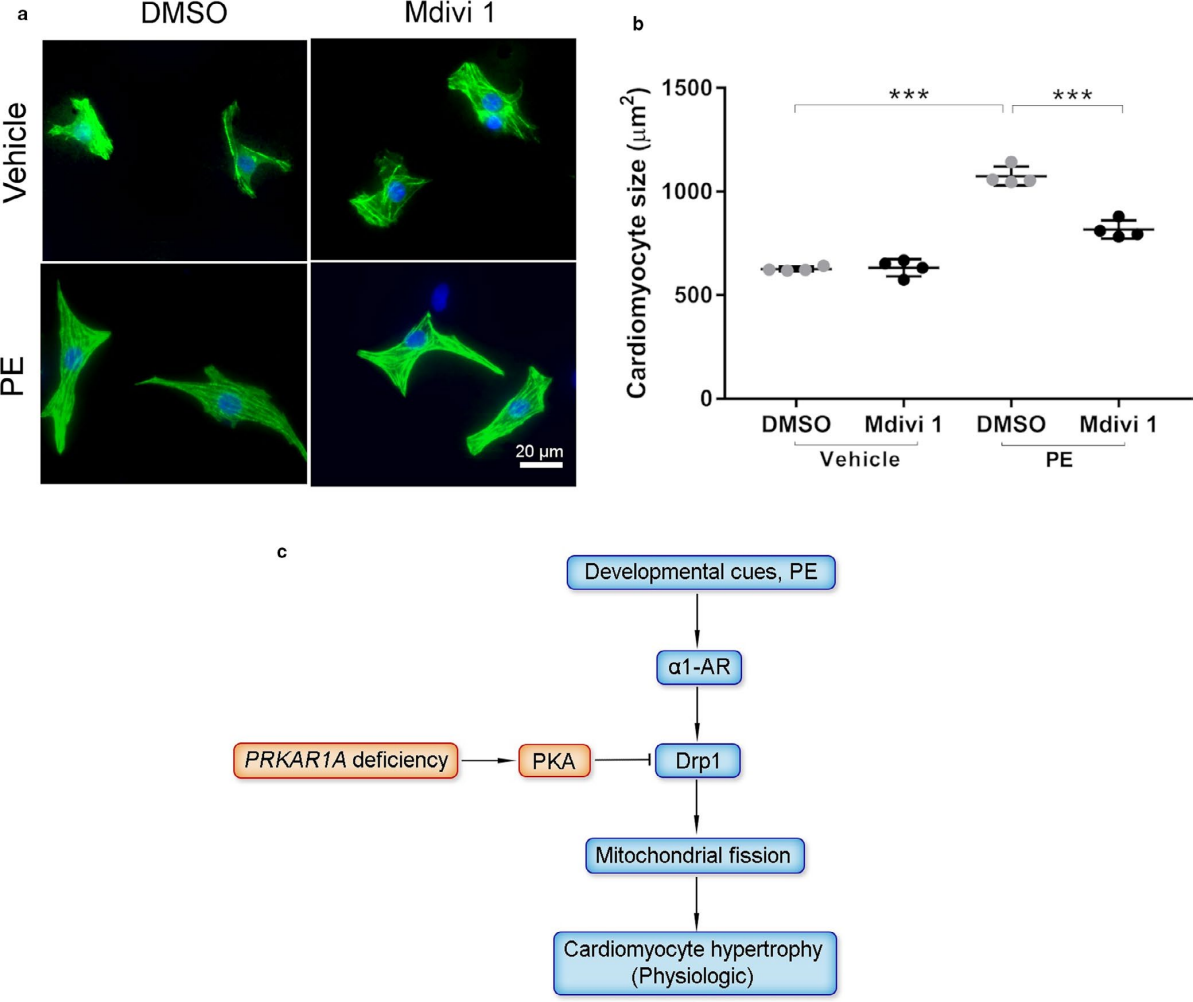
Inhibition of Drp1 attenuated α1‐adrenergic receptor‐mediated hypertrophy. (a,b) NRCMs were pretreated with the Drp1 inhibitor Mdivi 1 (50 µM) for 1h followed by incubation with PE (50 µM) for 48h. (a) Immunofluorescence staining for cTnT (*green*) and nuclei (DAPI, *blue*). Scale bar = 20 µm. (b) Cardiomyocyte cell surface area. Two‐way ANOVA with Sidak test. *** *p* < .001. (c) Schematic summary. Developmental cues initiate physiologic cardiac hypertrophy through α1‐adrenergic receptor (α1‐AR)‐mediated, Drp1‐dependent mitochondrial fission. *PRKAR1A* deficiency impedes hypertrophic heart growth, likely through PKA‐dependent Drp1 inactivation. Arrow, activation; bar‐headed line, inhibition

To determine if inhibition of Drp1 suppresses cardiomyocyte hypertrophy, NRCMs were pretreated with the Drp1 inhibitor Mdivi 1 followed by stimulation with PE. Treatment with Mdivi 1 attenuated PE‐induced hypertrophy (Figure 7a,b), suggesting that Drp1 activity is necessary for α1‐adrenergic receptor‐mediated cardiomyocyte hypertrophy.

## 4 DISCUSSION

Cardiac complications account for more than half of all deaths in patients with CNC, a familial multiple neoplasia syndrome caused by mutations of *PRKAR1A *(Correa et al., 2015). However, the impact of CNC on heart development remains unknown. In this study, we demonstrated that left ventricular mass was reduced in young CNC patients. Our animal studies further revealed that cardiac‐specific ablation of *PRKAR1A* impeded myocardial hypertrophic growth during development. Disruption of *PRKAR1A* provoked PKA‐dependent inhibition of the mitochondrial fission protein Drp1, a critical molecule necessary for initiation of cardiomyocyte hypertrophy (Figure 7c). Our results uncovered the effect of the naturally occurring *PRKAR1A* haploinsufficiency on heart morphology for the first time, and provided potential mechanistic explanations.

The reduction in heart mass in our *cPRKAR1A* ^+/‐^ mice was associated with a decrease in cardiomyocyte cross‐sectional area, suggesting that deletion of *PRKAR1A* hampered cardiomyocyte hypertrophy. It has been shown that cardiac‐specific homozygous ablation of *PRKAR1A* induces thinning of the ventricular walls at embryonic day 11.5, due to a significant reduction in cardiomyocyte proliferation (Yin et al., 2008). As the *Mlc2v‐Cre* ^+/‐^ line used in our study induces significant recombination as early as embryonic day 8.3 (Davis, Maillet, Miano, & Molkentin, 2012), the decreased heart weight in adult *cPRKAR1A* ^+/‐^ mice may also be attributed to diminished cardiomyocyte proliferation during early embryonic development. Interestingly, global *PRKAR1A* null embryos are smaller in size (Amieux et al., 2002), suggesting that *PRKAR1A* is likely universally required for growth of various tissues.

Accumulating evidence indicates that PKA catalytic activity is necessary for the phenotypes caused by *PRKAR1A* deficiency. In response to cAMP stimulation, tumors with *PRKAR1A* mutations collected from CNC patients exhibit higher total PKA activity than tumors from non‐CNC patients (Kirschner et al., 2000). Moreover, constitutive PKA activation has been detected in cardiac myxoma, a characteristic manifestation of CNC (Tseng et al., 2017). Homozygous deletion of *PRKAR1A* increases basal PKA activity, resulting in heart defects and embryonic lethality (Amieux et al., 2002; Yin et al., 2008). Interestingly, loss of *PRKACA* (encoding PKA catalytic subunit α) partially rescues the cardiac phenotype in global *PRKAR1A* null embryos (Amieux et al., 2002). Moreover, survival of the cardiac‐specific homozygous *PRKAR1A* knockout mice is extended to 18 weeks by *PRKACA* haploinsufficiency, but is only prolonged by 1 day by haploinsufficiency of *PRKACB* (encoding PKA catalytic subunit β) (Yin, Pringle, Jones, Kelly, & Kirschner, 2011). These results suggest that *PRKAR1A* deficiency predominantly activates the PKA catalytic subunit α. In this study, *PRKAR1A* deficiency modestly increased phosphorylation of certain PKA substrates in adult hearts (Figure 2a). PKA activity might be higher during early development of the *cPRKAR1A* ^+/‐^ hearts, as previously shown for the homozygous *PRKAR1A* knockout heart (Amieux et al., 2002; Yin et al., 2008). Indeed, prolonged PKA activation in adult mice can cause degradation of the PKA catalytic subunit, resulting in decreased total PKA activity (Hemmings, 1986). Moreover, PKA has been shown to activate phosphodiesterases, which mediate cAMP degradation and eventually restricts PKA activity (Liu et al., 2012).

Our in vivo and in vitro studies revealed that depletion of *PRKAR1A* attenuated cardiomyocyte hypertrophy, likely due to PKA activation. Indeed, PKA is known to inhibit cardiomyocyte hypertrophy through phosphorylation of histone deacetylase 4 (HDAC4) and HDAC5, which then accumulate in the nuclei and repress myocyte enhancer factor 2 (MEF2)‐dependent transcription of genes involved in cell differentiation and growth (Backs et al., 2011; Ha et al., 2010). Particularly, adrenergic stimulation‐induced expression of fetal genes, such as the classical hypertrophy markers atrial natriuretic peptide (ANP) and β‐myosin heavy chain (β‐MHC), is augmented by the PKA inhibitor H89, but is diminished by the PKA activators forskolin or 8‐CPT‐6‐Phe‐cAMP (Patrizio et al., 2008). Deficiency of phosphodiesterase 1C (PDE1C), a major phosphodiesterase that hydrolyzes cAMP in human myocardium, attenuates pathological cardiomyocyte hypertrophy in vitro and in vivo, in a PKA‐dependent manner (Knight et al., 2016). Conversely, inhibition of PKA with PKI restores hyperglycemia‐induced hypertrophy (Cheng et al., 2019). It is noteworthy that persistent PKA activation in a cardiac‐specific PKA catalytic subunit α transgenic mice results in cardiomyocyte hypertrophy and ventricular dilation (Antos et al., 2001). The discrepancy may be due to chronic exposure of the transgenic hearts to supraphysiological levels of PKA activity (~2.4‐ to 8‐fold increase vs. controls) (Antos et al., 2001). In cardiomyocytes, cAMP and PKA activity are not uniformly distributed but are confined primarily within the plasma membrane, sarcoplasmic reticulum, and myofilament compartments (Ghigo & Mika, 2019). Different extracellular stimuli induce differential activation of PKA within specific compartments (Liu et al., 2012). Therefore, aggravated hypertrophy of the PKA transgenic hearts may also be caused by nonselective activation of PKA in additional subcellular locales.

PKA phosphorylates Drp1 at S637, resulting in Drp1 inactivation and consequent inhibition of mitochondrial fission (Chang & Blackstone, 2007; Cribbs & Strack, 2007). Interestingly, Drp1‐mediated mitochondrial fission is necessary for norepinephrine‐induced cardiomyocyte hypertrophy (Pennanen et al., 2014). In this study, we showed that depletion of *PRKAR1A* provoked Drp1 S637 phosphorylation and inhibited Drp1‐mediated mitochondrial fission. Importantly, pharmacologic inhibition of Drp1 was sufficient to block PE‐induced hypertrophy. These studies suggest that *PRKAR1A* deficiency diminished cardiomyocyte hypertrophy in vitro, likely through inactivation of Drp1. It has been shown that loss of Drp1 activity suppresses cardiac hypertrophy and reduces skeletal muscle mass in vivo (Favaro et al., 2019; Hasan et al., 2018). Although phospho‐Drp1 (S637) was undetectable in the heart at 3 months of age (data not shown), it cannot be excluded that ablation of *PRKAR1A* might enhance Drp1 S637 phosphorylation during early cardiac development. Therefore, it remains possible that *PRKAR1A* deficiency suppresses myocardial hypertrophy also through inhibition of Drp1 in vivo.

Our clinical findings have limitations. Firstly, left ventricular mass in CNC patients was compared with pediatric normal references, which can be variable depending on the methodology used (Buechel, Kaiser, Jackson, Schmitz, & Kellenberger, 2009; Ven et al., 2020). An alternative control could potentially be age‐ and gender‐matched healthy siblings of the CNC patients. Secondly, our sample size is relatively small partly because CNC clinical cases are rare. These findings in patients need to be confirmed by larger clinical studies.

In conclusion, this study reveals that *PRKAR1A* deficiency delays heart growth during development. Mechanistically, *PRKAR1A* deficiency inhibits cardiomyocyte hypertrophy, likely through PKA‐dependent inhibition of Drp1. As *PRKAR1A* haploinsufficiency in humans causes CNC, our study may provide novel mechanistic insights regarding the cardiac mortality associated with this hereditary genetic disorder.

## CONFLICT OF INTERESTS

Dr. Stratakis' laboratory at the NIH holds patents on *PRKAR1A* and related genes and/or their function, and has received funding from Pfizer Inc. on research projects unrelated to the subject of this article.

## AUTHOR CONTRIBUTIONS

Y. L., P. X., and J. C. conducted the experiments and analyzed the data; Y. L. and Z. C. designed the research and wrote the article. W. P. B. and C. A. S. analyzed the human data and provided intellectual input; L. S. K. provided essential reagents and intellectual input. Z. C. conceived the hypothesis and supervised the project.
