# Supplementary material for: PRKAR1A deficiency impedes hypertrophy and reduces heart size
Source: Physiol Rep. 2020 Mar 25;8(6):e14405. doi: 10.14814/phy2.14405 (PMC7093752; doi:10.14814/phy2.14405)
Supplement: Supplementary file 1 [file PHY2-8-e14405-s001.pdf]

PRKAR1A deficiency impedes hypertrophy and reduces heart size

Yuening Liu[1], Peng Xia[1], Jingrui Chen[1], W. Patricia Bandettini[2], Lawrence S. Kirschner[3], Constantine A. Stratakis[4,#], Zhaokang Cheng[1,#]

[1] Department of Pharmaceutical Sciences, Washington State University, PBS 423, 412 E. Spokane Falls Blvd., Spokane, WA 99202-2131, USA
[2] National Heart, Lung, and Blood Institute, National Institutes of Health, 10 Center Drive, Rm B1D-47, Bethesda, Maryland 20892-1061, USA
[3] Department of Cancer Biology and Genetics, The Ohio State University, BRT 510, 460 W 12th Avenue, Columbus, Ohio 43210, USA
[4] Section on Endocrinology and Genetics, *Eunice Kennedy Shriver* National Institute of Child Health and Human Development, National Institutes of Health, 10 Center Drive, Building 10, NIH-Clinical Research Center, Room 1-3330, MSC1103, Bethesda, Maryland 20892, USA

Supplemental Materials

Supplemental Materials include 1 Supplemental Figure with legend.

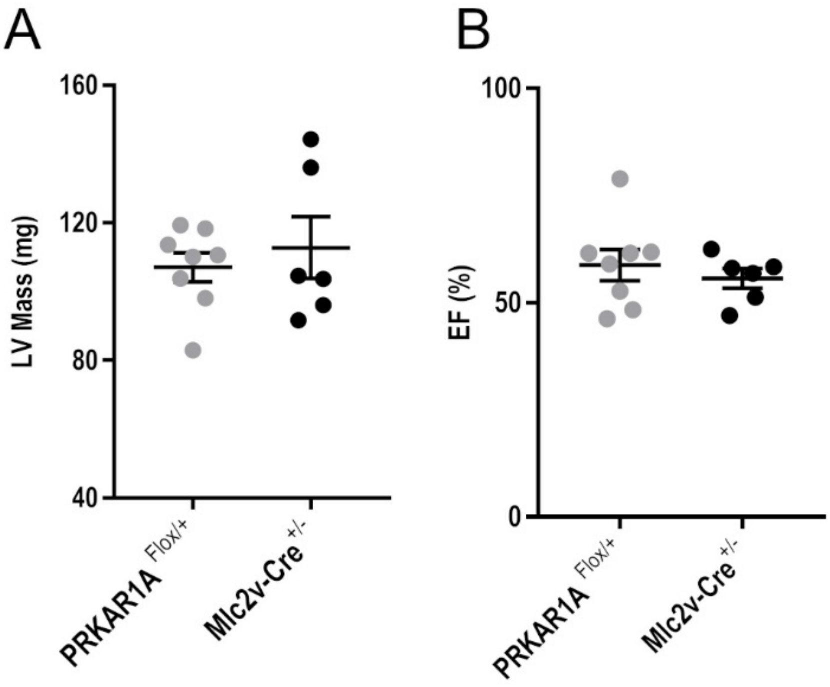

Supplemental Figure S1. Left ventricle mass and cardiac function were comparable between *PRKAR1A*$^{Flox/+}$ and *Mlc2v-Cre*$^{+/-}$ mice. **(A)** Left ventricular (LV) mass, and **(B)** LV ejection fraction (EF) was assessed using echocardiography. *PRKAR1A*$^{Flox/+}$, n=8; *Mlc2v-Cre*$^{+/-}$, n=6.
